# Supplementary material for: Data-based intervention approach for Complexity-Causality measure
Source: PeerJ Comput Sci. 2019 May 27;5:e196. doi: 10.7717/peerj-cs.196 (PMC7924450; doi:10.7717/peerj-cs.196)
Supplement: Text S1 — This file provides explanation of how dictionary construction is done for estimating conditional CCC for multi-variate measurements. A table for detailed description of how CCC values can be computed to be either positive or negative is included here. We also describe the criteria and rationale for choosing the parameters of CCC and details of our MATLAB implementation that is made available for free download and use. Additional results of testing of CCC on simulations which could not be accommodated in the main paper are included here. [file peerj-cs-05-196-s001.pdf]

Data-based intervention approach for Complexity-Causality measure
Supplementary Material

Aditi Kathpalia, Nithin Nagaraj

In this supplementary material, we provide details of our proposed method Compression-Complexity Causality (CCC), which are not covered in the main paper. We explain the way dictionary construction is done for estimating conditional CCC for multi-variate measurements. A table for detailed description of how CCC values can be computed to be either positive or negative is included here. We also describe the criteria and rationale for choosing the parameters of CCC and details of our MATLAB implementation that is made available for free download and use. Additional results of testing of CCC on simulations which could not be accommodated in the main paper are included here.

1 Dictionary building for conditional CCC

To estimate causality from time series Y to X, amidst the presence of other variables (say Z and W), two time varying dictionaries are built — D that encodes information from all variables (X, Y, Z, W) and D' that encodes information from all variables except Y (X, Z, W only). Suppose the time series blocks being considered at a time t are X_{past}, Y_{past}, Z_{past} and W_{past}, then the dictionary at that time D_{past} is built as follows. Suppose (for example)

$$\begin{pmatrix} X_{past} \\ Y_{past} \\ Z_{past} \\ W_{past} \end{pmatrix}$$

blocks of length 4 time points take values

$$\begin{pmatrix} 0 & 0 & 1 & 0 \\ 1 & 0 & 1 & 0 \\ 1 & 1 & 1 & 1 \\ 0 & 1 & 1 & 1 \end{pmatrix},$$

after each time series block (such as X_{past}) is binned using 2 bins. Then encoding in D_{past} is done based on assigning a particular value to each column. As each row in the first column can take 2 values, there exists a total of 16 possible combinations that the 4 rows can take together in a column. We encode information in 4 rows to a single row by assigning combinations of different values in the 4 rows an encoding from '0' to '15'. In the dictionary D_{past}, the above sequences are encoded as a single sequence —

$$\begin{pmatrix} 6 & 3 & 15 & 3 \end{pmatrix}.$$

The second dictionary D'_{past} at the same time constructed using all variables except Y similarly encodes blocks

$$\begin{pmatrix} X_{past} \\ Z_{past} \\ W_{past} \end{pmatrix}$$

taking values

$$\begin{pmatrix} 0 & 0 & 1 & 0 \\ 1 & 1 & 1 & 1 \\ 0 & 1 & 1 & 1 \end{pmatrix}$$

as

$$\begin{pmatrix} 2 & 3 & 7 & 3 \end{pmatrix}$$

assigning each column one particular state out of 8 possible states. Thus, for the above example, $D = (6, 3, 15, 3)$ and $D' = (2, 3, 7, 3)$. Effort-to-Compress (ETC) [1] can now be applied on the two dictionaries D and D' as these sequences are now just 1-dimensional symbolic sequences.

2 Positive and Negative CCC

CCC values estimated can be either positive or negative. This is because the dynamical compression-complexities estimated for the purpose of CCC estimation, $CC(\Delta X|X_{past})$ and $CC(\Delta X|X_{past}, Y_{past})$, can take either positive or negative values. How different cases result because of different signs of the above dynamical compression-complexities and their implication on CCC is discussed in Table S1.

3 Parameter selection for CCC: Criteria and Rationale

In Table S2, we summarize the criteria and rationale for choosing the four parameters (w, δ, B, L) of the proposed measure CCC. We have described the measure of Compression-Complexity Causality in the main paper with the idea of intervention. Appropriate parameter selection criteria is done with the view to find out the correct intervention point for a time series to check its causal influence on another given time series . Put more specifically, the main task is choosing the correct value for the length of the time series block Y_{past} and accordingly for X_{past}.

The parameter w which is the length of the moving window ΔX is fixed to 15 for all the datasets used in this work. It is chosen such that it contains sufficient number of data points over which CC rate can be reliably estimated. Earlier studies have revealed that ETC is able to reliably capture complexity of even very short time series (as small as length of 10 samples) [2]. δ, the step size by which the ΔX as well as X_{past} window is moved, is chosen based on the criteria of sufficient overlap $(20 - 50\%)$ between successive X_{past} windows of length L. B, the number of bins used to generate the symbolic sequence of the input time series is chosen such that it is sufficient to capture the underlying dynamics. It was found that for the AR processes, $B \geq 2$ is sufficient whereas the time series from the chaotic tent map requires at least $B = 8$.

Once w, δ, B are chosen, we choose L, the window length of X_{past}. For this, we analyze the curves of ETC measure as they vary with L, for different time series blocks as appropriate for a given dataset. A detailed description of selection criteria for L is discussed below.

3.1 Selection Criteria for L

As discussed in Table S2, for given time series X and Y, we first plot $ETC(X_{past} + \Delta X)$ and $ETC(Y_{past} + \Delta X)$ vs. L when causality is to be checked from Y_{past} to ΔX. We choose a value of L at which the two curves are well separated. In this work, we start with an $L = 20 (> w)$ and go up to $L = 300$ (in case of the predator prey ecosystem data, only 62 data points were available and thus we go up to $L = 40$). In Figures S1, S2, S3 and S4 which show these curves plotted for linearly and non-linearly coupled tent maps, predator prey and squid giant axon systems respectively, there exists some range of values of L for which the two curves are well separated. A value of L can thus be chosen from within this range. The choice of L for these curves is based on averaged ETC values for referred blocks over the entire time series. However, the choice of L may vary with time if we expect to have causality at different temporal scales with varying time. Moreover, for all the cases taken we have chosen the same values of L for checking causality from Y_{past} to ΔX and for checking causality from X_{past} to ΔY. These values can however be different depending on the curves of $ETC(X_{past} + \Delta X)$, $ETC(Y_{past} + \Delta X)$ and $ETC(Y_{past} + \Delta Y)$, $ETC(X_{past} + \Delta Y)$ respectively.

The separation between the curves $ETC(X_{past} + \Delta X)$ and $ETC(Y_{past} + \Delta X)$ is taken to give Y_{past} the maximum opportunity to cause ΔX. The complexities of these time series blocks will be very different at the scale at which there is an influence from past block of Y to the present block of X. Thus the choice of L is about adaptive determination of the temporal scale at which causality exists from Y to X.

Table S1: Sign of Dynamical Compression-Complexities, $CC(\Delta X|X_{past})$ and $CC(\Delta X|X_{past}, Y_{past})$, and their resulting implication on the sign of estimated Compression Complexity-Causality, $CCC_{Y_{past}\rightarrow\Delta X}$.

$CC(\Delta X\|X_{past}, Y_{past})$ / $CC(\Delta X\|X_{past})$	$-ve$	$+ve$
$-ve$	$X_{past} + \Delta X$ was more structured than X_{past}. Further, two cases arise. 1. When $\|CC(\Delta X\|X_{past})\| > \|CC(\Delta X\|X_{past}, Y_{past})\|$, $CCC_{Y_{past}\rightarrow\Delta X} < 0$. Here, intervention by Y_{past} in the joint case **degraded the structure** by bringing patterns different from X_{past}. Dynamical influence of Y_{past} on ΔX is very different from the dynamical influence of X_{past} on ΔX. e.g.: CCC from independent tent map to dependent tent map. 2. When $\|CC(\Delta X\|X_{past})\| < \|CC(\Delta X\|X_{past}, Y_{past})\|$, $CCC_{Y_{past}\rightarrow\Delta X} > 0$. Intervention by Y_{past} in the joint case **enhanced the structure** by bringing patterns similar to X_{past}. Dynamical influence of Y_{past} on ΔX is very similar to the dynamical influence of X_{past} on ΔX .e.g.: CCC from independent autoregressive (AR) process to dependent AR process.	$CCC_{Y_{past}\rightarrow\Delta X}$ is $-ve$ always. $X_{past} + \Delta X$ was more structured than X_{past}. Intervention by Y_{past} in the joint case **degraded the structure**. Dynamical influence of Y_{past} on ΔX is very different from the dynamical influence of X_{past} on ΔX. e.g.: CCC from independent tent map to dependent tent map.
$+ve$	$CCC_{Y_{past}\rightarrow\Delta X}$ is $+ve$ always. $X_{past} + \Delta X$ was less structured than X_{past}. Intervention by Y_{past} in the joint case **enhanced the structure** by bringing patterns similar to X_{past}. Dynamical influence of Y_{past} on ΔX is very similar to the dynamical influence of X_{past} on ΔX.	$X_{past} + \Delta X$ was less structured than X_{past}. Further, two cases arise. 1. When $\|CC(\Delta X\|X_{past})\| > \|CC(\Delta X\|X_{past}, Y_{past})\|$, $CCC_{Y_{past}\rightarrow\Delta X} > 0$. Here, intervention by Y_{past} in the joint case **enhanced the structure** by bringing patterns similar to X_{past}. Dynamical influence of Y_{past} on ΔX is very similar to the dynamical influence of X_{past} on ΔX. 2. When $\|CC(\Delta X\|X_{past})\| < \|CC(\Delta X\|X_{past}, Y_{past})\|$, $CCC_{Y_{past}\rightarrow\Delta X} < 0$. Intervention by Y_{past} in the joint case **degraded the structure** by bringing patterns different from X_{past}. Dynamical influence of Y_{past} on ΔX is very different from the dynamical influence of X_{past} on ΔX .

Table S2: Criteria and rationale for choosing the parameters (w, δ, B, L) for CCC. Values of each parameter chosen for Autoregressive (AR), Tent Map (TM), Squid Giant Axon System (SA) and Predator Prey Ecosystem (PP) are enlisted in the rightmost column. Please refer to the main paper for details of these four systems.

**Parameter**	**Description**	**Criteria**	**Rationale**	**Values Chosen**
w	Window length ΔX	Minimal data length over which CC rate can be reliably estimated.	Earlier studies have revealed that ETC is able to reliably capture complexity of even very short time series [2].	AR: 15<br>TM: 15<br>SA: 15<br>PP: 15
δ	Step-size	An overlap of $20-50\%$ between successive time series windows (X_{past} of length L) over which CC is estimated.	To capture the continuity of time series dynamics.	AR: 80<br>TM: 80<br>SA: 50<br>PP: 4*
B	Number of bins	Smallest number of symbols that capture the time series dynamics.	CCC requires symbolic sequences that represent the underlying dynamics.	AR: 2<br>TM: 8<br>SA: 2<br>PP: 8
L	Window length of immediate past to ΔX (X_{past}) and (Y_{past})	After choosing w, δ, B as above, to check causal influence from Y_{past} to ΔX, we plot $ETC(X_{past} + \Delta X)$ and $ETC(Y_{past} + \Delta X)$ vs. L.<br>***First criteria***: Choose a value of L at which the two curves are well separated. If the above criteria fails (there is an overlap in the ETC curves for all L), we plot $ETC(X_{past}, Y_{past})$ and $ETC(X_{past} + \Delta X, Y_{past} + \Delta X)$ vs. L.<br>***Second criteria***: Choose a value of L such that the two curves are well separated.	Well separation of the complexity values of time series blocks $(X_{past} + \Delta X)$ and $(Y_{past} + \Delta X)$ is taken to give maximum possible opportunity to Y_{past} to influence ΔX as against X_{past}. This L is hence the best intervention point. If no such value of L can be found, the maximum separation of curves (X_{past}, Y_{past}) and $(X_{past} + \Delta X, Y_{past} + \Delta X)$, gives the maximum opportunity to (X_{past}, Y_{past}) jointly to affect ΔX.	AR: 150<br>TM: 100<br>SA: 75<br>PP: 40

*This was an exception with 90% overlap as very short data length was available.

If the above criteria fails (there is an overlap in the curves), it means that at no temporal scale can Y intervene to make visible its dynamical influence on ΔX (by change of complexity) as against the dynamical influence due to past of X. We then plot $ETC(X_{past}, Y_{past})$ and $ETC(X_{past} + \Delta X, Y_{past} + \Delta X)$ vs. L. We choose a value of L such that the two curves are well separated. In case of AR processes where the first criteria is not met due to the overlap between $ETC(X_{past} + \Delta X)$ and $ETC(Y_{past} + \Delta X)$, the second pair of curves is plotted as shown in Figure S5. The rationale behind this criteria is to see at which intervention point L do X_{past}, Y_{past} jointly begin to have an influence on the dynamical evolution of ΔX.

If the two time series are independent or are constant in time and identical, both the above criteria are bound to fail. This implies that there exists no temporal scale at which there is an influence from one of these time series to the other. For the case of two independent and uniformly distributed real time series the curves for both criteria are shown in Figures S7 and S8. There exists no value of L at which there is a causality from Y to X or vice versa.

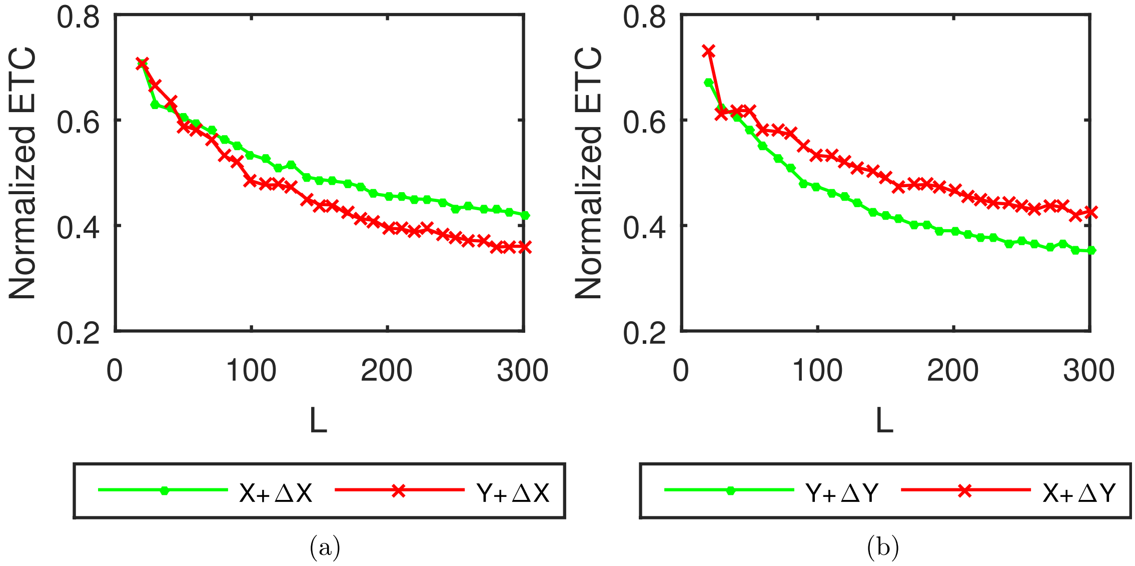

Figure S1: (color online). Averaged $ETC(X_{past} + \Delta X)$, $ETC(Y_{past} + \Delta X)$ curves in subfigure (a) and $ETC(Y_{past} + \Delta Y)$, $ETC(X_{past} + \Delta Y)$ curves in subfigure (b) for linearly coupled tent maps ($\epsilon = 0.2$) with Y causing X (simulated as per Eq. 17, 18 of the main paper). $w = 15, \delta = 100, B = 8$ and L is incremented by a value of 5 data points each time. Using the first criteria for selection of L, $L = 100$ to 300.

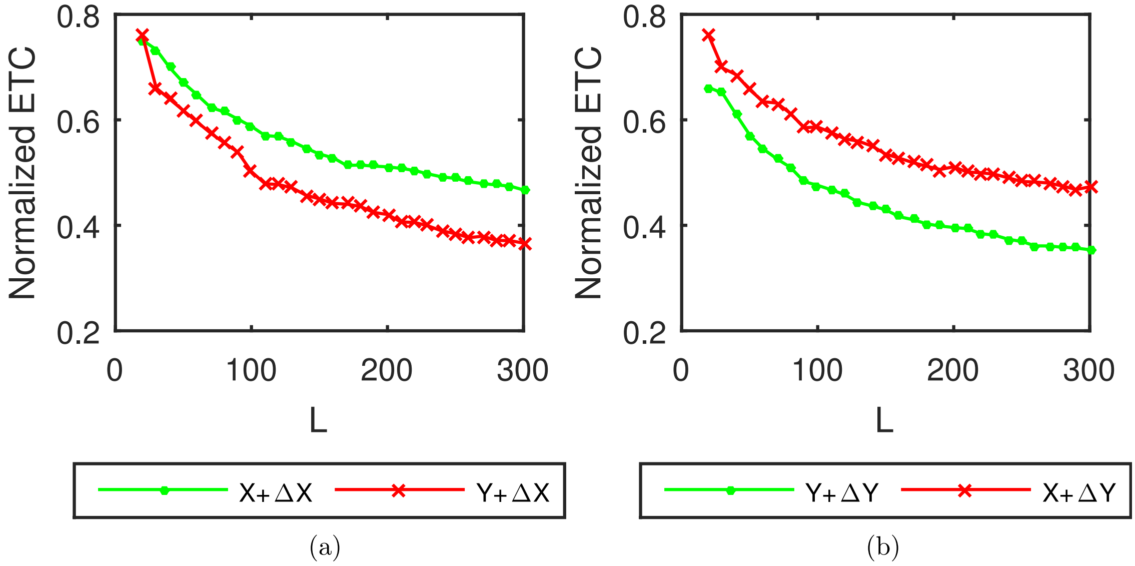

Figure S2: (color online). Averaged $ETC(X_{past} + \Delta X)$, $ETC(Y_{past} + \Delta X)$ curves in subfigure (a) and $ETC(Y_{past} + \Delta Y)$, $ETC(X_{past} + \Delta Y)$ curves in subfigure (b) for non linearly coupled tent maps ($\epsilon = 0.2$) with Y causing X (simulated as per Eq. 1, 2). $w = 15, \delta = 100, B = 8$ and L is incremented by a value of 5 data points each time. Using the first criteria for selection of L, $L = 75$ to 300.

4 Results

Some additional results for the proposed measure CCC and its comparison with existing methods, Transfer Entropy (TE) and Granger Causality (GC), are given below.

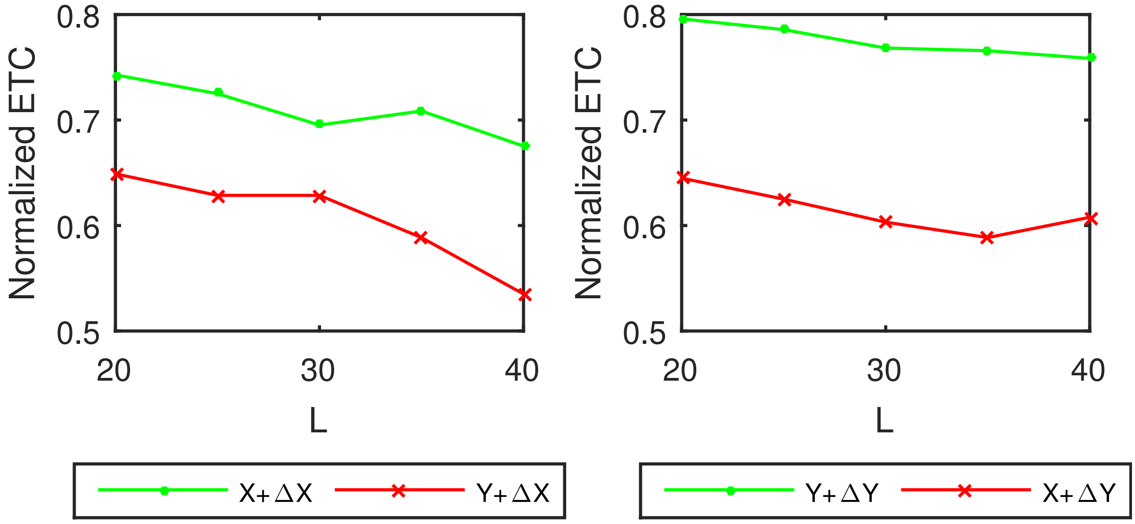

(a) (b)

Figure S3: (color online). Averaged $ETC(X_{past} + \Delta X)$, $ETC(Y_{past} + \Delta X)$ curves in subfigure (a) and $ETC(Y_{past} + \Delta Y)$, $ETC(X_{past} + \Delta Y)$ curves in subfigure (b) for predator prey ecosystem with Y representing Didinium (predator) population and X representing Paramecium (prey) population. $w = 15, \delta = 1, B = 8$ and L is incremented by a value of 5 data points each time. Using the first criteria for selection of L, $L = 20$ to 40.

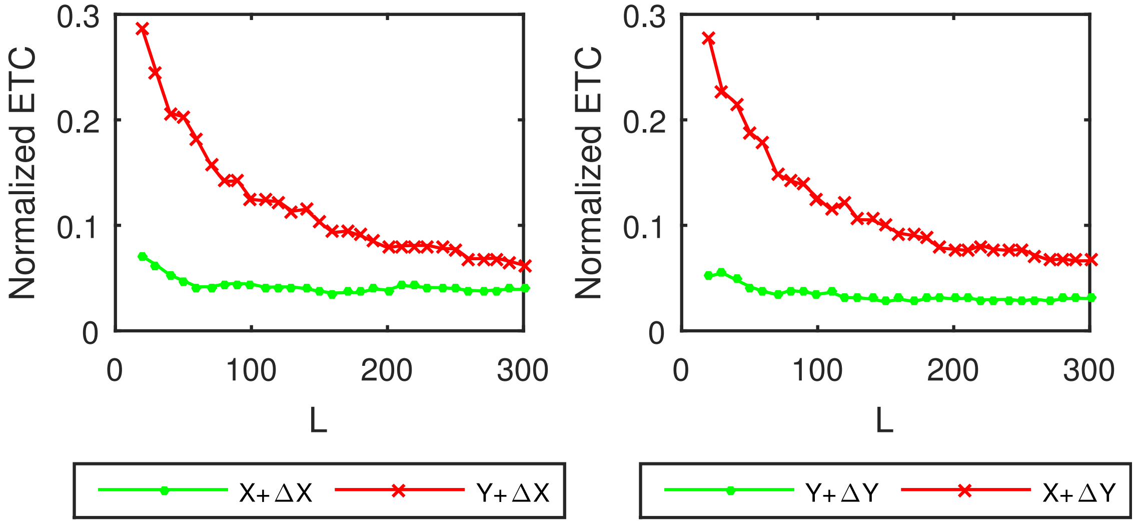

(a) (b)

Figure S4: (color online). Averaged $ETC(X_{past} + \Delta X)$, $ETC(Y_{past} + \Delta X)$ curves in subfigure (a) and $ETC(Y_{past} + \Delta Y)$, $ETC(X_{past} + \Delta Y)$ curves in subfigure (b) for squid giant axon system ('a5t01') with Y representing the applied stimulus current and X representing observed voltage. $w = 15, \delta = 100, B = 2$ and L is incremented by a value of 5 data points each time. Using the first criteria for selection of L, $L = 75$ to 300. Lower values of L are not used despite sufficient separation so as to avoid making computation based on the transient stage values.

4.1 Non-linearly coupled tent maps

Non-linearly coupled tent maps were simulated as per the following equations. Independent process, Y, is generated as:

$$\begin{aligned} Y(t) &= 2Y(t-1), \qquad 0 \leq Y(t-1) < 1/2, \\ Y(t) &= 2 - 2Y(t-1), \qquad 1/2 \leq Y(t-1) \leq 1. \end{aligned} \tag{1}$$

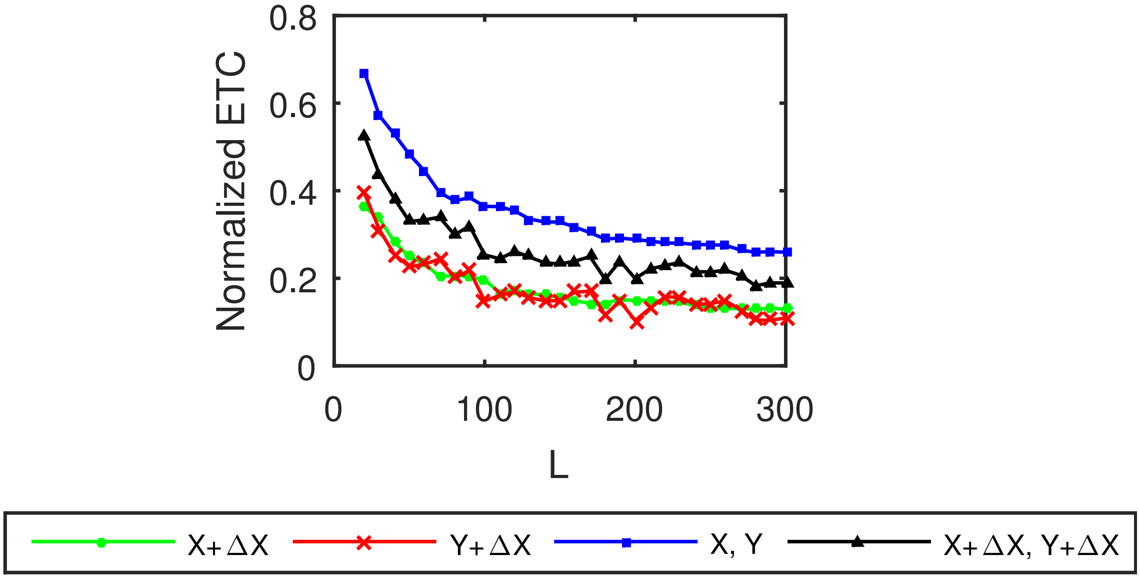

Figure S5: (color online). Averaged $ETC(X_{past} + \Delta X)$, $ETC(Y_{past} + \Delta X)$, $ETC(X_{past}, Y_{past})$, $ETC(X_{past}+\Delta X, Y_{past}+\Delta X)$ curves for coupled AR processes with Y causing X (simulated as per Eq. 15 with all settings as in Section 5.1.1 of the main paper with $\epsilon = 0.8$). $w = 15, \delta = 100, B = 2$ and L is incremented by a value of 5 data points each time.Using the second criteria for selection of L, $L = 100$ to 300.

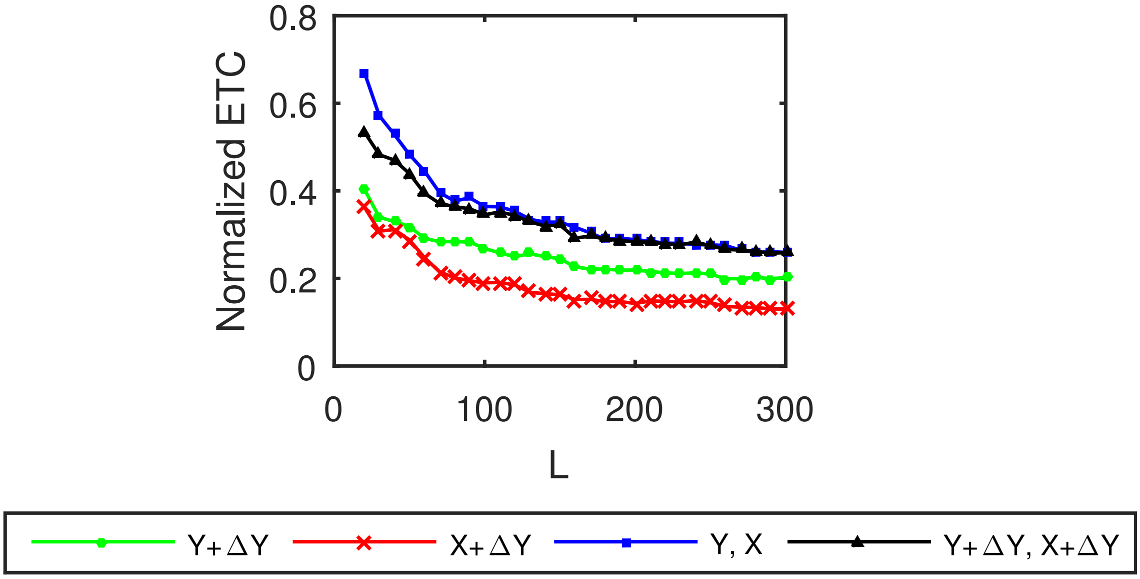

Figure S6: (color online). Averaged $ETC(Y_{past} + \Delta Y)$, $ETC(X_{past} + \Delta Y)$, $ETC(Y_{past}, X_{past})$, $ETC(Y_{past}+\Delta Y, X_{past}+\Delta Y)$ curves for coupled AR processes with Y causing X (simulated as per Eq. 15 with all settings as in Section 5.1.1 of the main paper with $\epsilon = 0.8$). $w = 15, \delta = 100, B = 2$ and L is incremented by a value of 5 data points each time. Using the first criteria for selection of L, $L = 100$ to 300.

The dependent process, X, is as below:

$$\begin{aligned} X(t) &= 2f(t), & 0 \leq f(t) < 1/2, \\ X(t) &= 2 - 2f(t), & 1/2 \leq f(t) \leq 1, \\ f(t) &= \epsilon Y(t-1) + (1-\epsilon)X(t-1), \end{aligned} \tag{2}$$

where ϵ is the degree of non-linear coupling.
The length of the signals simulated in this case was 3000, i.e. $t = 1$ to 3000s, sampling period

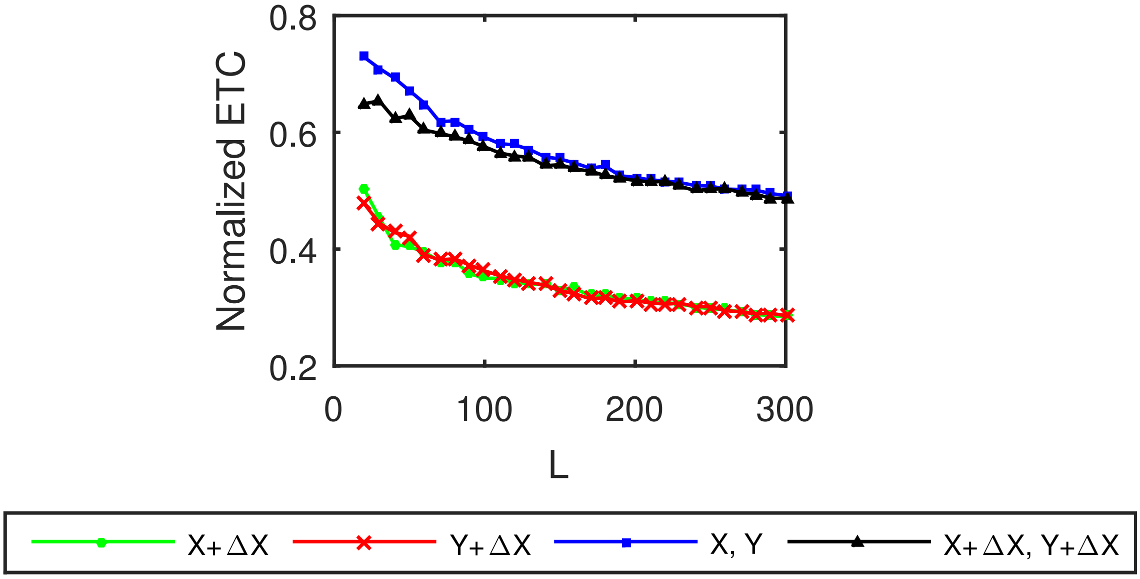

Figure S7: (color online). Averaged $ETC(X_{past} + \Delta X)$, $ETC(Y_{past} + \Delta X)$, $ETC(X_{past}, Y_{past})$, $ETC(X_{past} + \Delta X, Y_{past} + \Delta X)$ curves for independent processes Y and X. $w = 15, \delta = 100, B = 2$ and L is incremented by a value of 5 data points each time.

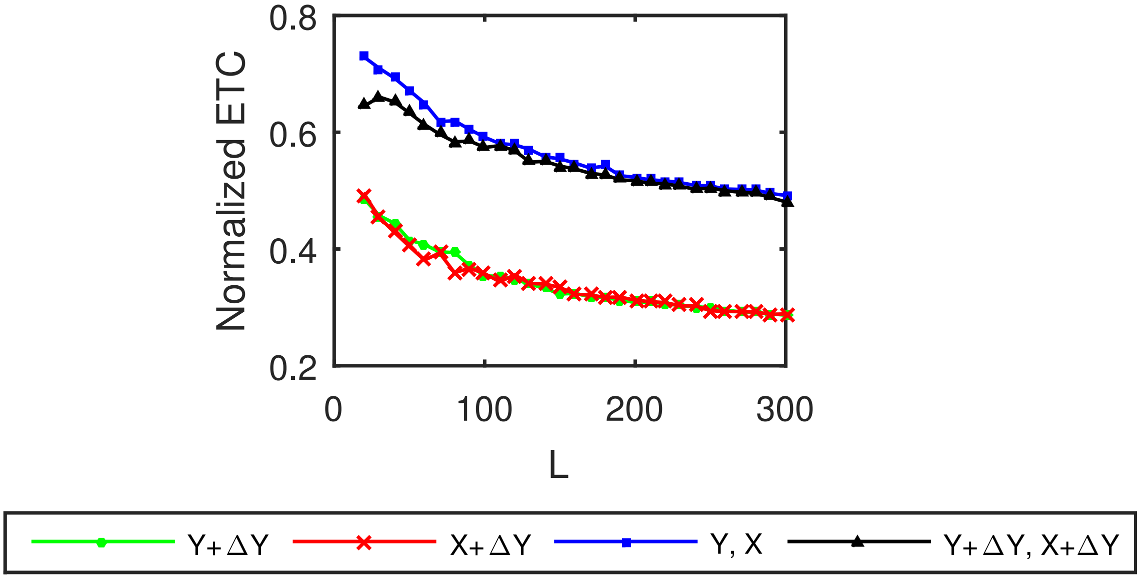

Figure S8: (color online). Averaged $ETC(Y_{past} + \Delta Y)$, $ETC(X_{past} + \Delta Y)$, $ETC(Y_{past}, X_{past})$, $ETC(Y_{past} + \Delta Y, X_{past} + \Delta Y)$ curves for independent processes Y and X. $w = 15, \delta = 100, B = 2$ and L is incremented by a value of 5 data points each time. Using the second criteria for selection of L, based on this figure and Fig. S7, $L = 100$ to 300, avoiding the range of L giving transient values of CCC.

$=$ 1s and the first 2000 transients were removed to yield 1000 points for causality estimation. Figure S9 shows the performance of CCC and TE for non-linearly coupled tent maps (as mean values over 50 trials) as ϵ is varied (CCC settings: $L = 100$, $w = 15$, $\delta = 80$, $B = 8$). The assumption of a linear model for estimation of GC was proved to be erroneous for most trials and hence GC values are not displayed.

As ϵ is increased for both linear and non-linear coupling, $TE_{Y \to X}$ increases in the positive direction and then falls to zero when the two series become completely synchronized at $\epsilon = 0.5$. The trend of the magnitude of CCC values is similar to TE, however, $CCC_{Y \to X}$ increment is in negative direction. This is because of the fact that with increasing coupling the kind of dynamical

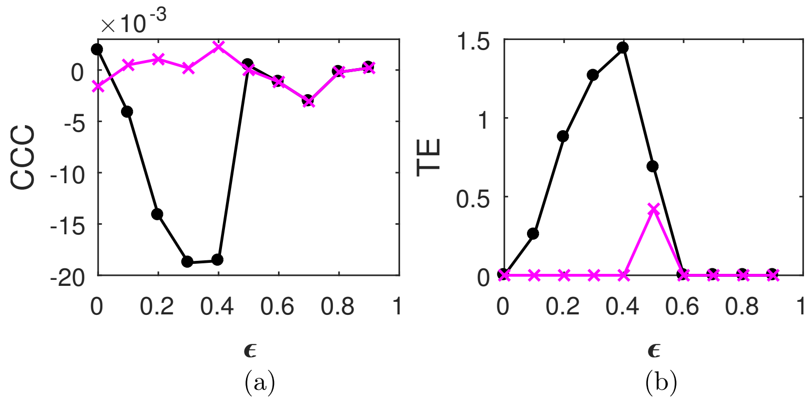

Figure S9: (color online). Mean of causality values estimated using CCC (a) and TE (b) for non-linearly coupled tent maps, from Y to X (solid line-circles, black) and X to Y (solid line-crosses, magenta) as the degree of coupling is increased. With increasing coupling (until synchronization), magnitude of CCC and TE values increases. CCC values are negative while TE are positive.

influence from Y to X becomes increasingly different than the dynamical influence from the past values of X to itself.

The range of standard deviation of CCC values from Y to X is 0.0057 to 0.0087 for different values of ϵ and that from X to Y is 0.0057 to 0.0102. For TE, Y to X, standard deviation range is 0 to 1.2854 and X to Y, standard deviation range is 0 to 1.0479.

Kullback-Leibler divergence (KL) and Jensen Shannon Divergence (JSD) were also estimated along with CCC to verify increasing negative values of CCC obtained with increasing non-linear coupling between tent maps. These results are shown in Figure S10.

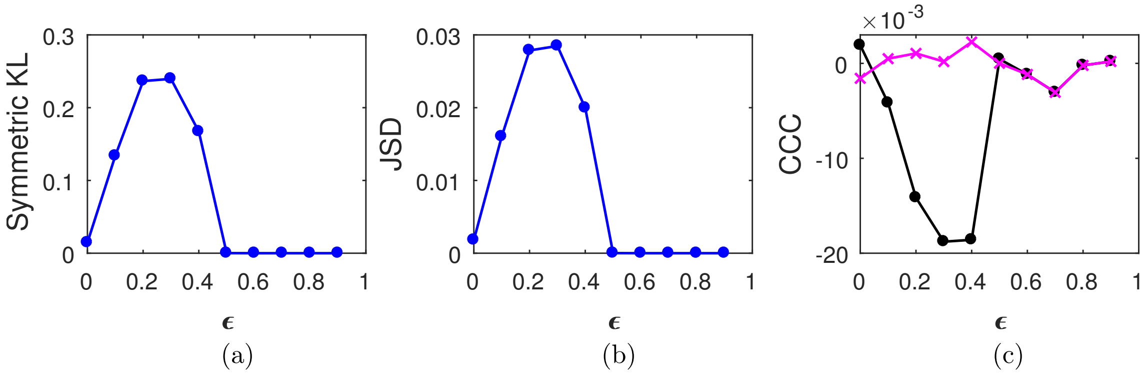

Figure S10: (color online). Mean values of divergence between distributions of non-linearly coupled tent maps using Symmetric Kullback Leibler (KL) (a) and Jensen Shannon (JSD) (b) divergences (in nats), and the mean of causality values estimated using CCC from Y to X (solid line-circles, black) and X to Y (solid line-crosses, magenta) (c), as the degree of coupling, ϵ is varied. For $\epsilon < 0.5$, CCC and KL/JSD are highly negatively correlated.

4.2 Decimated coupled signals with uniform sampling

It is often the case that the rate of sampling of acquired measurements is not equal to the rate of generation of the process. Causal inferences are regularly made from such data [3], for e.g., fMRI signals [4, 5] as well as other neurophysiological recordings [6], climate data [7]. Two sets of coupled AR processes were first simulated and subsequently decimated.

Set 1 of AR processes, of order 1, were simulated as below:

$$\begin{aligned} Y(t) &= 0.7Y(t-1) + \varepsilon_{Y,t}, \\ X(t) &= 0.9X(t-1) + 0.8Y(t-1) + \varepsilon_{X,t}. \end{aligned} \tag{3}$$

Set 2 of AR processes, of order 5, were simulated as below:

$$\begin{aligned} Y(t) &= 0.7Y(t-5) + \varepsilon_{Y,t}, \\ X(t) &= 0.9X(t-5) + 0.8Y(t-1) + \varepsilon_{X,t}, \end{aligned} \tag{4}$$

where, noise terms, $\varepsilon_Y, \varepsilon_X = \nu\eta$, where ν = noise intensity = 0.03 and η follows standard normal distribution. The original length of X and Y simulated in both the sets is 2000. Upon decimation, the length of the time series reduces. β represents the decimation factor that scales the sampling frequency. As β is varied from 1 to 0.5, sampling frequency is scaled from its original value to half its value.

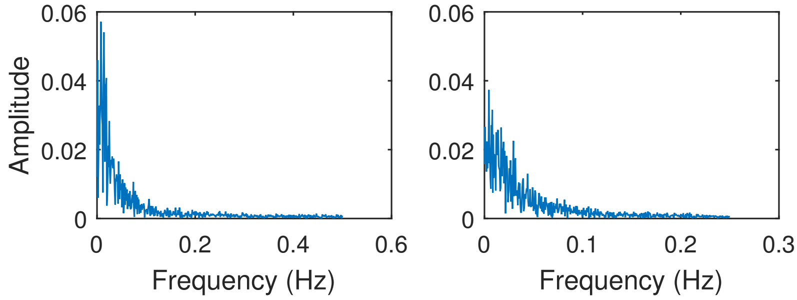

(a) (b)

Figure S11: Frequency Spectrum of dependent AR(1) process from *Set 1* without decimation (a) and when decimation factor equals 0.5 (b). The process does not undergo aliasing on decimation.

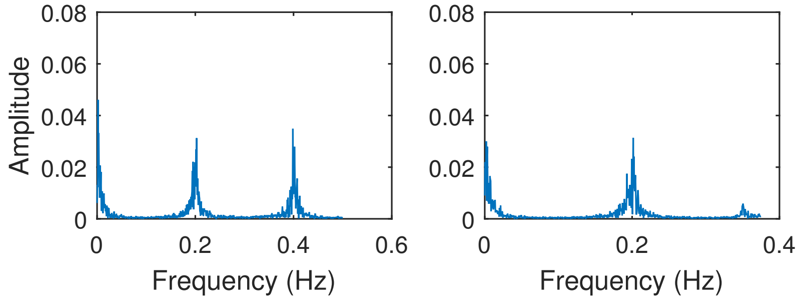

(a) (b)

Figure S12: Frequency Spectrum of of dependent AR(5) process from *Set 2* without decimation (a) and when decimation factor equals 0.75 (b). The process undergoes aliasing on decimation.

Set 1 of processes, being of order one, have low frequency components in the signal. As a result, even when β is reduced to 0.5, it does not lead to frequency folding in the spectrum of process Y and X. Frequency spectrum for a trial of X in this process is shown in Figure S11 for the original case and the case where β is reduced to 0.5. In case of *Set 2*, decimation of the signals Y and X leads to aliasing. This is because higher frequency components are present in the signals, leading to folding of these frequencies even as β is reduced to 0.8. The frequency spectrum for a trial of X for its non-decimated version and for decimation with β equal to 0.75 is shown in Figure S12.

4.2.1 Equal decimation of independent and dependent signal

When both signals Y and X from the two sets are decimated by scaling their sampling rate by an equal decimation factor, β, ranging from 1 to 0.5 at intervals of 0.05, the results obtained using the three methods, CCC, TE and GC are as shown in Figures S13 and S14. Figure S13 shows the results for *Set 1* while Figure S14 shows results for *Set 2* as mean causality values estimated over 10 trials. CCC settings for both sets: $L = 150$, $w = 15$, $\delta = 80$, $B = 2$.

While the values of CCC are relatively consistent even upon decimation (with or without aliasing), those of TE and GC are stable only in case of non-aliased decimation. For GC and TE in the aliased case, even though there is no confounding of the causality direction, the magnitude of

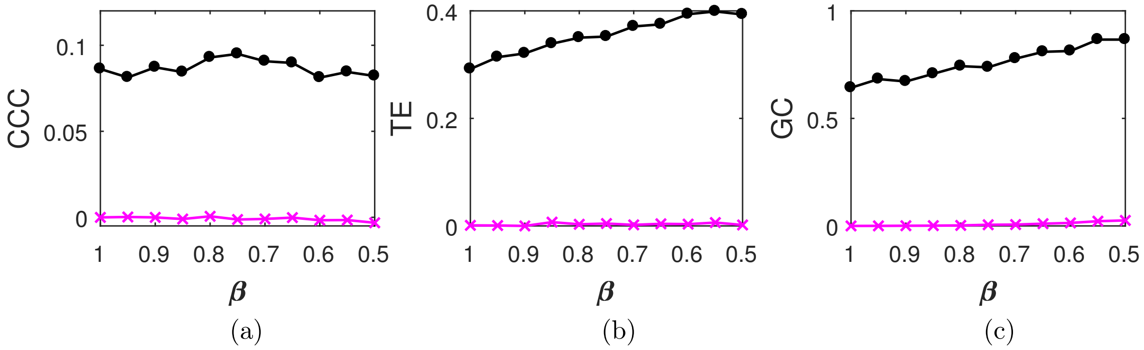

Figure S13: (color online). Mean causality estimated using CCC (a), TE (b) and GC (c) for coupled AR processes from Y to X (solid line-circles, black) and X to Y (solid line-crosses, magenta) as the decimation factor β is varied for both independent and dependent signal. The coupled AR processes simulated do not undergo frequency aliasing. All three measures perform fairly well in this case.

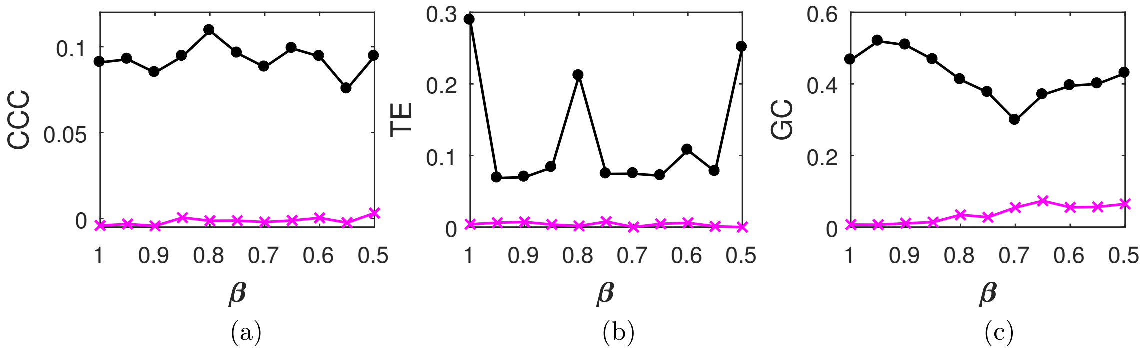

Figure S14: (color online). Mean causality estimated using CCC (a), TE (b) and GC (c) for coupled AR processes from Y to X (solid line-circles, black) and X to Y (solid line-crosses, magenta) as the decimation factor β is varied for both independent and dependent signal. The coupled AR processes simulated become frequency aliased. CCC values are stable compared to TE and GC.

causality estimated is not consistent and reliable.

4.2.2 Decimation of dependent signal

When only signal X is decimated by scaling its sampling rate by a decimation factor β, ranging from 1 to 0.5 at intervals of 0.05, the results obtained using the three methods CCC, TE and GC are as shown in Figures S15 and S16. Figure S15 shows the results for *Set 1* while Figure S16 shows results for *Set 2* as mean causality values estimated over 10 trials. For the length of the two signals to match, the independent signal considered is truncated at the length of the dependent signal. CCC settings for both sets: $L = 150$, $w = 15$, $\delta = 80$, $B = 2$.

In this scenario, for both non-aliased and aliased decimation, CCC estimates are much more stable and consistent (across β) when compared to those of TE and GC, where confounding in the direction of causality results even upon slightest decimation. It is clear from these results that CCC is the most robust, reliable and consistent among the three causality measures.

5 Description of CCC Toolbox

The accompanying CCC toolbox, implemented in MATLAB contains the following files:

1. **demo_2processes.m** calls functions to simulate a system of two coupled AR processes or tent maps to estimate the value of Compression-Complexity Causality between them.

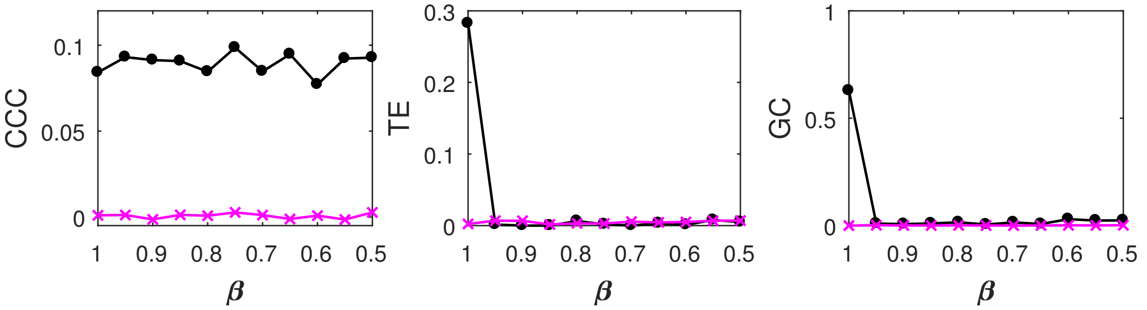

(a) (b) (c)

Figure S15: (color online). Mean causality estimated using CCC (a), TE (b) and GC (c) for coupled AR processes from Y to X (solid line-circles, black) and X to Y (solid line-crosses, magenta) as the decimation factor β is varied for the dependent signal. The dependent AR process simulated does not undergo frequency-aliasing. Only CCC can capture the correct direction and strength of coupling when β is decreased.

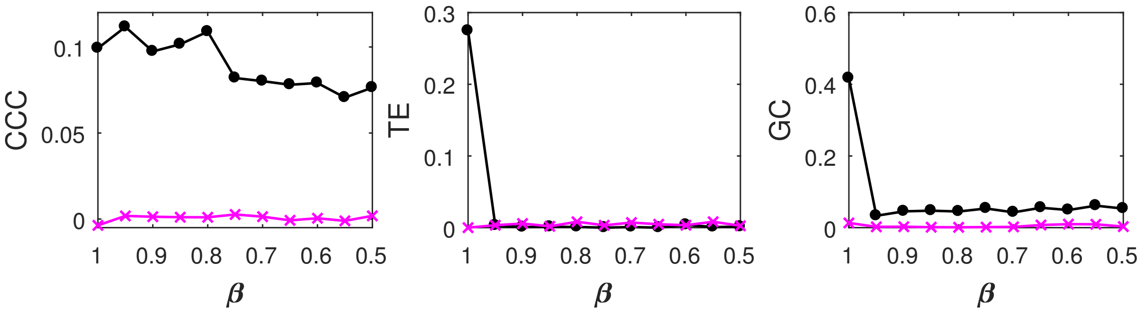

(a) (b) (c)

Figure S16: (color online). Mean causality estimated using CCC (a), TE (b) and GC (c) for coupled AR processes from Y to X (solid line-circles, black) and X to Y (solid line-crosses, magenta) as the decimation factor is varied for the dependent signal. The dependent AR process simulated becomes frequency aliased. Only CCC can capture the correct direction and strength of coupling when β is decreased.

2. **demo_3processes.m** calls functions to simulate a system with three AR processes with coupling between them and estimates the value of conditional Compression-Complexity Causality between any two variables chosen.
3. **coupled_AR.m** simulates a system of two unidirectionally coupled AR processes with a desired level of noise or percentage of non-uniform sampling.
4. **puncture.m** introduces non-uniform sampling/non-synchronous measurements in the data.
5. **coupled_tent.m** simulates a system of two unidirectionally non-linearly coupled tent maps.
6. **UpdateTent.m** updates the values of the tent map at every iteration.
7. **coupled_AR_3processes.m** simulates a system of three coupled AR processes.
8. **conditional_CCC.m** estimates conditional Compression-Complexity Causality between any two input variables (time series) from a given multivariate system.
9. **ETC.m** estimates individual/joint ETC values. Dn_to_D1.m subroutine called by the ETC function performs the task of dictionary building.
10. **Partition.m** bins the given time series before estimating ETC values.

References

[1] N. Nagaraj, K. Balasubramanian, and S. Dey, "A new complexity measure for time series analysis and classification," *The European Physical Journal Special Topics*, vol. 222, no. 3-4, pp. 847–860, 2013.

[2] K. Balasubramanian and N. Nagaraj, "Aging and cardiovascular complexity: effect of the length of RR tachograms," *PeerJ*, vol. 4, p. e2755, 2016.

[3] D. Smirnov and B. Bezruchko, "Spurious causalities due to low temporal resolution: Towards detection of bidirectional coupling from time series," *EPL (Europhysics Letters)*, vol. 100, no. 1, p. 10005, 2012.

[4] G. H. Glover, "Overview of functional magnetic resonance imaging," *Neurosurgery Clinics*, vol. 22, no. 2, pp. 133–139, 2011.

[5] S.-G. Kim, W. Richter, and K. Uğurbil, "Limitations of temporal resolution in functional mri," *Magnetic resonance in medicine*, vol. 37, no. 4, pp. 631–636, 1997.

[6] I. M. de Abril, J. Yoshimoto, and K. Doya, "Connectivity inference from neural recording data: Challenges, mathematical bases and research directions," *Neural Networks*, 2018.

[7] A. Moberg, D. M. Sonechkin, K. Holmgren, N. M. Datsenko, and W. Karlén, "Highly variable northern hemisphere temperatures reconstructed from low-and high-resolution proxy data," *Nature*, vol. 433, no. 7026, p. 613, 2005.
